# Support by telestroke networks is associated with increased intravenous thrombolysis and reduced hospital transfers: A german claims data analysis

**DOI:** 10.1186/s13561-024-00577-5

**Published:** 2024-11-28

**Authors:** Andreas Janßen, Nicolas Pardey, Jan Zeidler, Christian Krauth, Jochen Blaser, Carina Oedingen, Hans Worthmann

**Affiliations:** 1 Leibniz University Hannover, Hannover, Germany; 2 Center for Health Economics Research Hannover (CHERH), Hannover, Germany; 3 Institute for Epidemiology, Hannover Medical School, Social Medicine and Health Systems Research, Hannover, Germany; 4 Techniker Krankenkasse (Health Insurance)-Representative Office of Lower Saxony, Hannover, Germany; 5 Erasmus School of Health Policy & Management, Erasmus University Rotterdam, Rotterdam, The Netherlands; 6 Department of Neurology, Hannover Medical School, Carl-Neuberg-Straße 1, 30623 Hannover, Germany

**Keywords:** Stroke, Telestroke network, Teleneurology, Claims data analysis, Statutory health insurance

## Abstract

**Background:**

Acute stroke treatment is time-critical. To provide qualified stroke care in areas without 24/7 availability of a stroke neurologist, the concept of teleneurology was established, which is based on remote video communication through telemedicine organized by telestroke networks. Data on the effectiveness and efficiency of stroke treatment via teleneurology is very scarce and is therefore partly questioned in the healthcare sector. The aim was to evaluate stroke care in hospitals with and without teleneurology in Northern Germany.

**Methods:**

We conducted a retrospective case–control data analysis using health insurance claims data for the years 2018 to 2021. Based on pre-defined criteria, two models were defined and clinical as well as health economic parameters were compared. In model 1, we compared patients from hospitals with and without support by a telestroke network, while in model 2, we compared patients from hospitals with and without support by a telestroke network, including only districts without a certified stroke unit. Assessed parameters were age, length of stay, patients’ comorbidities, inpatient costs, reasons for discharge, qualified stroke care treatment according to operation and procedure codes (OPS) and intravenous thrombolysis (IVT) rates.

**Results:**

Hospitals supported by a telestroke network improved their rate of stroke care according to OPS and increased more than three-fold their IVT rate (*p* = 0.042). In comparison, patients from hospitals with support by a telestroke network had a higher number and rate of qualified stroke care according to OPS (model 1: 73.6% vs 2.2%, *p* < 0.001 and model 2: 57.0% vs 3.8%, *p* < 0.001), higher rate of IVT (model 1: 9.5% vs. 0.0%, *p* = 0.027 and model 2: 10.3% vs 0.0%, *p* = 0.056) and a lower rate of secondary transfers to another hospital (model 1: 5.9% vs. 28.9%, *p* < 0.001 and model 2: 5.6% vs 30.1%, *p* < 0.001). Inpatient costs were lower in cases treated in hospitals with support by a telestroke network (model 1: 4,476€ vs. 5,549€, *p* = 0.03 and model 2: 4,374€ vs. 5,309€, *p* = 0.02). In multivariate analysis costs were independently associated with length of stay and patient transfer to another hospital but not with support by a telestroke network.

**Conclusion:**

Hospitals with support by a telestroke network are associated with improved qualified stroke care resulting in higher rates of IVT and stroke care according to OPS codes as well as lower rates of onward transfers. Costs per patient were independently associated with transfer rates and length of hospital stay.

**Supplementary Information:**

The online version contains supplementary material available at 10.1186/s13561-024-00577-5.

## Background

Stroke is the second leading cause of disability and death worldwide [1]. The annual incidence of stroke and stroke-related death increased worldwide between 1990 and 2021 [2]. Stroke is also associated with high costs. A recent study showed that approximately $393 billion, which is about 0.3% of the total global GDP, was associated with costs of treatment, rehabilitation, social care and informal care of stroke in 2017 [3]. The burden of stroke is a common problem in Germany, and demographic change is exacerbating the problem. In Germany, approximately 250,000 strokes occur annually. A study, which used statutory health insurance billing data for calculation, stated a 1-year prevalence of 317 cases per 100,000 inhabitants for the year 2007 [4].

During an acute untreated large ischemic stroke up to 1.9 million neurons die every minute and the brain ages by 3.6 years per hour [5]. Accordingly, acute recanalizing stroke care consisting of intravenous thrombolysis (IVT) and endovascular treatment (EVT) is time-critical urging the need for organized acute stroke care [6, 7]. The in-hospital treatment of stroke patients should take place in accordance with a stroke unit (SU) concept [8]. This includes monitoring of vital signs, blood pressure and heart rhythm, early treatment of complications and neurological deficits and optimised diagnostics for decision-making for secondary prevention. In Germany, currently 314 certified neurological SU are available [9]. They provide the best possible stroke care but are resource-intensive requiring a number of qualified personal. Consequently, some regions in Germany have persistent low coverage by SU [10]. However, it is discussed that poor SU coverage is related to regional structural weakness. The alternative is treatment in the next hospital close to the patients’ home without a specialized SU and no 24/7 availability of a stroke neurologist denying the patient the required qualified stroke treatment.

To fill this gap, the concept of teleneurology was developed, based on teleconsultation via remote video transmission, ensuring immediate qualified neurological examination, even while the patient is in the emergency room. Teleneurology is considered as a technology-intensive, but resource-saving method to provide expertise to local hospitals and to ensure acute treatment [11]. The first German telestroke network was implemented in Southern Germany in 2001 [12]. Teleneurological stroke treatment has continued to grow in Germany, resulting in 225 hospitals offering stroke treatment supported by a telestroke network adjacent to a comprehensive stroke center (CSC) [13]. As a result, currently every 10th stroke patient in Germany receives telemedical care [14]. Despite SU concepts and partially teleneurologically supported SU treatments, there are still regions without sufficient stroke treatment availability. In these regions, patients do not have access to specialized treatment, leading to an increased burden on patients’ life as well as on the healthcare system. In Germany, data showing the extent to which teleneurology improves stroke care is limited. Nevertheless, in 2003 an advantage for telemedicine has been shown in the state of Bavaria where acute stroke care improved in hospitals supported by a telestroke network [15]. Nevertheless, the benefits of teleneurology for stroke treatment are partly questioned in the healthcare system e.g. by payers, so that further data is needed. The aim of the current study is to evaluate stroke care in hospitals with and without support by a telestroke network in the state of Lower Saxony, Germany by comparison of rates of qualified stroke care according to OPS, specialized therapy using IVT, transfer rates and treatment costs.

## Methods

### Study population

We used pseudonymized statutory health insurance claims data from the Techniker Krankenkasse (TK) Lower Saxony (~ 930,000 insurees) between 2018 and 2021 to investigate teleneurological stroke care of the teleneurology network of a tertiary care center (Hannover Medical School teleneurology network: MHH TNN) and affiliated hospitals. The dataset contained both first and subsequent hospital stays from 2018 to 2021. Only cases with either a primary discharge or secondary diagnosis of stroke or transient ischemic attack (TIA) were selected. TIA patients were explicitly included, as they are comparable to the group of ischemic or hemorrhagic stroke in terms of cardiovascular risk and risk of recurrence. In many patients, a TIA cannot yet be differentiated from an infarction at the time of symptoms and should be treated at a stroke unit according to the guidelines of the German Neurological Society [8]. All cases of patients were excluded which were under 18 years of age, had a hospital stay > 365 days, were treated prior to 2018 and after 2021, had no assigned hospital or were cases from the hospitals supported by the telestroke network prior to establishing the support (2 hospitals affected).

Patient’s characteristics as well as quality indicators such as rates of achieved stroke care according to operation and procedure codes (OPS) and IVT as specialised treatment for acute ischemic stroke were collected. Rates of stroke care according to OPS refers to the following codes 8-980 (basic intensive-care treatment); 8-98f (complex intensive-care treatment); 8-981 (SU treatment); 8-98b (Other complex multimodal treatment: Other complex neurological treatment of acute stroke) issued by the German Federal Institute for Drugs and Medical Devices existing in the German diagnosis-related-group system [16, 17]. Secondly, patients treated in hospitals with support by a telestroke network were compared at the hospital and district level to hospitals without SU or support by a telestroke network and their according districts (see description of models 1 and 2 in the next paragraph). The study population included all insured persons of TK with inpatient ICD-10-GM diagnosis codes I60: subarachnoid hemorrhage, I61: intracerebral hemorrhage, I62: other nontraumatic intracranial hemorrhage, I63: cerebral infarction, I64: stroke not classified as hemorrhage or infarction and G45: transient cerebral ischemic attack (TIA) and related syndromes.

### Study Design

#### Models for comparison of patients from hospitals with and without support by telestroke networks

In the design of a retrospective descriptive study we selected patients from hospitals supported by the telestroke network MHH TNN to compare them with patients from eligible non-supported hospitals (controls) in Lower Saxony for analysis after applying predetermined categories of comparability in regard to each hospital’s care structure (see appendix Table 1). Based on underlying information about each hospital's care structures in 2021, hospitals with similar care structures were selected as controls. Therefore, for each hospital, detailed information was collected to map the care structure of the regions and the hospitals to identify relevant hospitals that could be considered for comparison of treated stroke patients. Final selection of hospitals was made prior to data analysis. Selection parameters are the number and type of hospital beds (hospital plan of Lower Saxony), the distance to the next certified regional SU and to the next CSC as well as the number of stroke cases between 2016 and 2020 (quality report of hospitals) [18, 19]. Additionally, structural prerequisites including availability of emergency care and CT-scanner 24/7, department of internal medicine and intensive care unit for every hospital were reviewed. The number and type of beds allow an estimation of the size and equipment of the hospitals. Comparing hospitals with significantly different numbers of beds could mean that hospitals have different resources and staff. The distance to the next certified SU and to the next CSC assumes similar prehospital emergency medical services referral concepts e.g. including emergency medical services (EMS) bypass systems. In dependency of the distance to the next SU, the patient is either transferred to the next hospital (with or without support by a telestroke network) or is directly transferred to the SU. Another important criterion is the number of strokes or TIAs treated at each hospital. Selected hospitals were required to have a total minimum of 100 stroke cases between 2016 and 2020. Hospitals were selected for two different models (model 1 and model 2).

In the first approach, stroke care is compared at the hospital level in hospitals without a certified SU. Therefore, patients from hospitals supported by a telestroke network were compared with patients from hospitals without support by a telestroke network (model 1). Of note, for each hospital supported by a telestroke network a comparable control hospital was selected. The aim was to include the highest possible number of valid comparisons in order to use a relatively large number of patients for the comparison. For each included hospital supported by a telestroke network one hospital without support by a telestroke network according to the above mentioned selection criteria was detected. There was one exception, in which two hospitals were potential controls for one of the hospitals with support by a telestroke network. Therefore, we performed a sensitivity analysis in which we selected the other hospital in question and repeated the comparative analyses. In the second approach, patients from hospitals supported by a telestroke network were compared to patients from hospitals without support by a telestroke network at the district level (model 2). Here, hospitals with support by a telestroke network and as controls hospitals without support by a telestroke network were selected, including only hospitals from districts without a certified SU (model 2). Accordingly, an emergency medical services bypass system directly to a certified SU cannot be deployed in a larger proportion of stroke patients. Thus, it seems coherent that the primary stroke care in the studied districts takes place at least to a large extent in the studied hospitals and not outside the districts, which emphasizes the importance of the analysed hospitals for stroke care. In this model, eligible hospitals in appropriate districts that met the selection criteria were included. For each district, we selected the most suitable hospitals as hospitals supported by a telestroke network or as control hospitals. In both models, clinical and health economic parameters from patients such as age, DRG weight (determining the revenue amount of a case), the duration of mechanical ventilation, the length of stay, the patient clinical complexity level (i.e., defined as the patient-related overall severity), and the costs of hospitalization were considered. In addition, reasons for discharge from hospital that included death, transfer to another hospital, discharge to a rehabilitation facility, and discharge to a nursing facility were evaluated.

To classify each hospitals’ quality of stroke care, the rate of stroke care according to OPS (OPS 8-98b, 8-980, 8-981 and 8-98f) and rate of IVT (OPS 8-020.8) were recognized (see appendix Table 2). Hospitals supported by telestroke networks use OPS code 8-98b “other neurological complex treatment of acute stroke”.

Furthermore, if cases were transferred to another hospital, transfer costs were assessed via case pooling and then compared. All transferred cases were combined with their subsequent stay and the costs were added up. All cases were compared between those treated in hospitals with and without support by a telestroke network. From a health economic perspective as a comparative cost-cost analysis the relation between costs per patient, teleneurology care and other potentially associated parameters were analysed.

#### Statistical analysis

The development of the rate of stroke care according to OPS and treatment with IVT between 2018 and 2021 was tested using the Jonckheere-Terpstra test. For comparisons of hospitals with support by a telestroke network and hospitals without support by a telestroke network Mann–Whitney-U test was used for continuous and Chi-Square test for categorical variables. A multiple linear regression analysis was performed to assess the relation between costs per patient and teleneurology care, length of stay, transfer to another hospital and discharge to a rehabilitation center (last variable only included in model 1 and not in model 2). The level of significance was set at p ≤ 0.05. The statistical software package IBM SPSS Statistics 26 was used for data analysis.

## Results

### Descriptive statistics

**Fig. 1:**
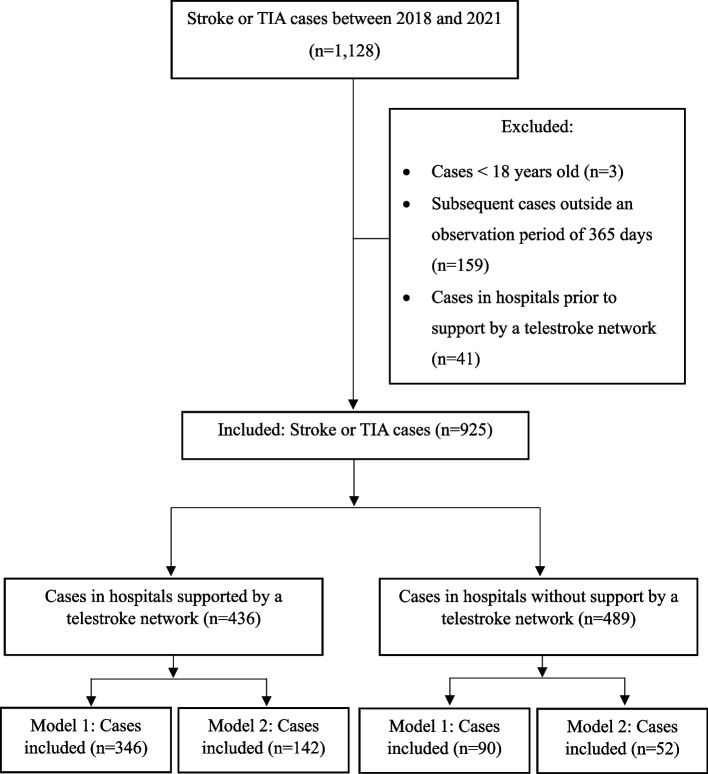
Selection of patients’ cases with stroke or TIA cases from the insurees of the TK Cases were identified with the following criteria: Initial hospitalization and subsequent stays with diagnosis ICD-10-GM G45, I60, I61, I62, I63, I64. Abbreviations: TIA, Transient ischemic attack. TK, Techniker Krankenkasse

The dataset contained 1,128 cases. Based on the criteria used to identify the study population, 436 cases from hospitals supported by a telestroke network and 489 cases from hospitals without support by a telestroke network between 2018 and 2021 were included (Fig. 1).

Regarding only patients from hospitals supported by a telestroke network the descriptive statistics can be seen in Table 1.

**Table 1:** Descriptive statistics for hospitals supported by a telestroke network between 2018 and 2021

Cases of hospitals supported by a telestroke network (*n* = 436 cases)	
Age [in years], median (IQR)	76 (63–82)
Length of stay [in days], median (IQR)	5 (3–8)
DRG weight, median (IQR)	0.95 (0.74–1.19)
Ventilation [in hours], median (IQR)	0 (0–0)
PCCL, median (IQR)	0 (0 – 2)
Billing amount [in euro], median (IQR)	4,056 (3,251 – 5,495)
OPS 8-98b (%)	41
OPS in total (%)	58
Death (%)	3.4
Transfer to another hospital (%)	6.7
Discharge to a rehabilitation facility (%)	6.2
Discharge to a nursing facility (%)	3.4

Continuous data are presented as median (interquartile range) and discrete data as proportions. OPS 8-98b includes all cases with stroke care according to OPS code 8-98b, OPS in total includes all cases with stroke care according to OPS codes 8-980, 8-98f, 8-981, 8-98b. Death, transfer to another hospital, discharge to a rehabilitation facility and discharge to a nursing facility describe reasons for discharge that have been documented for each case

*Abbreviations: IQR* Interquartile range, *OPS* Operation and procedure code, *PCCL* Patient clinical complexity level

**Fig. 2:**
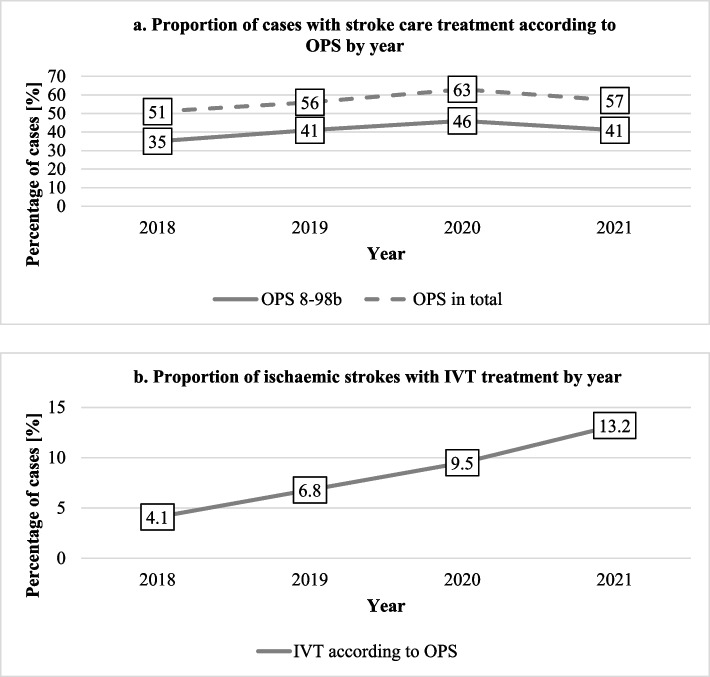
Rates of Stroke care according to OPS and IVT supported by a telestroke network over time Stroke care according to OPS 8-98b is defined as “Other neurological complex treatment of acute stroke “, Stroke care according to “OPS in total” includes all cases with documented OPS codes 8-980, 8-98f, 8-981, 8-98b. IVT rate includes all cases with documented OPS code 8-020.8 (Systemic thrombolysis). Abbreviation: IVT, Intravenous thrombolysis. OPS, Operation- and procedure code

Further, the rates of stroke care according to OPS codes 8-98b, 8-980, 8-981 and 8-98f and rate of IVT from the hospitals supported by a telestroke network were analysed from 2018 to 2021 (Fig. 2).

In 2018, the proportion of stroke care according to OPS 8-98b was 35% (*n* = 29), increased over the next two years and peaked in 2020 with a proportion of 46% (*n* = 58) and a proportion of 41% (*n* = 53) in 2021 (Fig. 2A). In 2018, 51% (*n* = 43) of all cases received stroke care according to at least one OPS code (8-98b, 8-980, 8-981, 8-98f). This proportion was 57% (*n* = 74) in 2021. However, proportions did not differ statistically (p > 0.05).

In 2018, the share of IVT performed in patients with AIS was 4.1% (*n* = 2) (Fig. 2B). The IVT rate rose steadily in the following years to a proportion of 13.2% (*n* = 10) in 2021 (comparison of IVT rate 2018 to 2021: *p* = 0.042).

### Comparison of patients from hospitals with and without support by a telestroke network

In model 1, patients were grouped according to hospitals with and without support by a telestroke network, with both groups consisting of patients from seven hospitals each. From hospitals with support by a telestroke network *n* = 346 cases and from hospitals without support by a telestroke network *n* = 90 cases were included (Table 2).

**Table 2:** Comparison of stroke cases in hospitals with or without support by a telestroke network between 2018 and 2021

	**Model 1**			**Model 2**		
	**(1)** **(*n* = 346)**	**(2)** **(*n* = 90)**	***p-value***	**(3)** **(*n* = 142)**	**(4)** **(*n* = 52)**	***p-value***
Age [in years], median (IQR)	75 (62–82)	79 (74–84)	**0.001**	77 (61–83)	79 (72–85)	0.093
Length of stay [in days], median (IQR)	5 (3–7)	6 (2–14)	0.249	5 (3–6)	6 (1–13)	0.878
DRG weight, median (IQR)	0.94 (0.74–1.19)	0.77 (0.34–1.18)	**0.021**	0.93 (0.73–1.14)	0.77 (0.32–1.08)	**0.021**
Ventilation hours [in hours], median (IQR)	0	0	0.836	0	0	0.808
PCCL, median (IQR)	0 (0–1)	0 (0–1)	0.596	0 (0–2)	0 (0–1)	0.697
OPS 8-98b^a^ (%)	52.5	2.2	** < 0.001**	53.5	0.03	** < 0.001**
OPS in total^a^ (%)	73.6	2.2	** < 0.001**	57.0	0.03	** < 0.001**
Death^a^ (%)	3.3	3.3	0.941	3.5	15.4	**0.004**
Transfer to another hospital^a^ (%)	5.9	28.9	** < 0.001**	5.6	30.1	** < 0.001**
Discharge to a rehabilitation facility^a^ (%)	4.5	3.3	0.671	2.1	1.9	0.935
Discharge to a nursing facility^a^ (%)	2.7	7.8	**0.020**	2.8	0	0.223

Continuous data are presented as median (interquartile range) and discrete data as proportions. Statistically significant results are shown in bold, *p* < 0.05 was considered significant. OPS 8-98b includes all cases with documented OPS code 8-98b, OPS in total includes all cases with documented OPS codes 8-980, 8-98f, 8-981, 8-98b. Death, transfer to another hospital, discharge to a rehabilitation facility and discharge to a nursing facility describe reasons for discharge that have been documented for each case

*Abbreviations: IQR* Interquartile range, *OPS* Operation and procedure code, *PCCL* Patient clinical complexity level. Group description: (1) = Hospitals supported by a telestroke network (2) = Hospitals not supported by a telestroke network, (3) = Hospitals supported by a telestroke network in districts without certified SU, (4) = Hospitals without support by a telestroke network in districts without SU

Patients from hospitals supported by a telestroke network showed higher rates of stroke care according to OPS 8-98b (52.5% vs 2.2%; *p* < 0.001) and in total stroke care according to OPS codes 8–980, 8-98f, 8–981 and 8-98b (73.6% vs 2.2%; *p* < 0.001), while rates of transfer to another hospital (5.9% vs 28.9%; *p* < 0.001) and discharge to a nursing facility (2.7% vs 7.8%; *p* = 0.020) were lower. However, patients in the hospitals supported by a telestroke network were slightly younger (75 vs 79 years; *p* = 0.001) and the DRG weight was higher (0.94 vs. 0.77; *p *< 0.021).

We also compared the number of ischemic strokes with IVT treatment between hospitals with and without support by a telestroke network. The hospitals with support by a telestroke network performed IVT in 9.5% (*n* = 19) of the cases, while the hospitals without support by a telestroke network performed IVT in 0.0% of the cases (*p* = 0.027) (not shown in Table 2).

### Comparison of patients in districts with and without support by a telestroke network

In model 2, only districts without hospitals with certified SU or known support by any possible telestroke network were observed. Patients from hospitals with and without support by a telestroke network were compared (Table 2). Hospitals with support by a telestroke network (from three districts) had a case number of *n* = 142 with a median age of 77 years and hospitals without support by a telestroke network (from seven districts) had a case number of *n* = 52 with a median age of 79 years. Again, this comparative approach shows differences between the groups. Patients from districts without support by a telestroke network showed a lower rate of stroke care according to OPS 8-98b (3.8% vs 53.5%; *p* < 0.001) and rate of stroke care according to OPS codes 8-980, 8-98f, 8-981 and 8-98b (3.8% vs. 57.0%; *p* < 0.001). The rate of transfers was also higher in the districts without support by a telestroke network (30.1% vs. 5.6%; *p* < 0.001). Additionally, higher proportion of cases died in the districts without support by a telestroke network (15.4% vs. 3.5%; *p* = 0.004). The DRG weight (0.93 vs. 0.77; *p* = 0.021) was higher in hospitals supported by a telestroke network.

Finally, the number of IVT performed for all ischemic strokes had a share of 10.3% (*n* = 9) in hospitals with support by a telestroke network, while in hospitals without support by a telestroke network no IVT was performed (*p* = 0.056) (not shown in Table 2).

### Transfer costs

Costs per patient with related transfers were compared for model 1 and model 2 (Table 3). The Mann–Whitney-U test showed significantly lower median costs for the telestroke-supported groups (model 1: 4,476€ vs. 5,549€; *p* = 0.03 and model 2: 4,374 vs. 5,309; *p* = 0.02). Comparison concluded all inpatient costs, including possible transfer costs to another hospital.

**Table 3:** Comparison of costs of stroke cases in hospitals with or without support by a telestroke network between 2018 and 2021

	**Model 1**			**Model 2**		
	**(1)** **(*n* = 346)**	**(2)** **(*n* = 90)**	***p-value***	**(1)** ***n* = 142**	**(2)** ***n* = 52**	***p-value***
Costs [in Euro], median (IQR)	4,476 (3,539–6,460)	5,549 (3,564–8,548)	**0.03**	4,374 (3,558 – 5,872)	5,309 (3,927 – 8,691)	**0.02**
mean (SD)	6,266 (6,682)	8,335 (11,903)		5,656 (6,192)	7162,78 (8,865)	

Data are presented as median (interquartile range) and mean (standard deviation). Statistically significant results are shown in bold, *p* < 0.05 was considered significant

*Abbreviations: IQR* Interquartile range, *SD* Standard Deviation. Group description: (1) = Hospitals supported by a telestroke network in districts without certified SU, (2) = Hospitals without support by a telestroke network in districts without SU

In a univariate analysis using Spearman rank correlation for metric and Mann–Whitney-U test for dichotomous variables, length of stay (model 1: *p* < 0.001; model 2: *p* < 0.001), transfer to another hospital (model 1: *p* < 0.001; model 2: *p* < 0.001), discharge to a rehabilitation facility (model 1: *p* < 0.001) and teleneurology as a dummy variable assigning a teleneurology case (model 1: *p* = 0.016; model 2: *p* = 0.009) were significantly associated with the costs per patient, while age (model 1: *p* = 0.454; model 2: *p* = 0.990, death (model 1: *p* = 0.657; model 2: *p* = 0.476), discharge to a rehabilitation facility (model 2: *p* = 0.305) or discharge to a nursing facility (model 1: *p* = 0.080; model 2: *p* = 0.235) were not associated. Thereafter, a multivariate model was applied to examine for independent association with costs per patient.

The multiple linear regression, with the covariates length of stay, transfer to another hospital, discharge to a rehabilitation facility (only model 1) and teleneurology revealed that length of stay and transfer to another hospital are significantly associated with higher costs, whereas teleneurology is not significantly associated with higher costs (model 1: length of stay: b: 474, 95%CI: 368–580, *p* < 0.001; transfer to another hospital: b: 13,600, 95%CI: 11,676–15,524, *p* < 0.001; discharge to a rehabilitation facility: b: 853, 95%CI: −2196–3902, *p* = 0.583; teleneurology: b: 1,455, 95%CI: −85–2,997, *p* = 0.064; model 2: length of stay: b: 456, 95%CI: 296–617, *p* < 0.001; transfer to another hospital: b: 9,967, 95%CI: 7,199–12,735, *p* < 0.001; teleneurology: b: 1,495, 95%CI: −527–3,517, *p* = 0.147).

### Sensitivity analysis: comparison of patients from hospitals with and without support by a telestroke network

Two suitable hospitals could be considered as controls for one hospital with support by a telestroke network in Model 1. Therefore, we repeated the analysis and included the other control hospital. The results can be seen in Table 3 and Table 4 from the appendix. From hospitals with support by a telestroke network *n* = 346 cases and from hospitals without support by a telestroke network *n* = 136 cases were included. Patients from hospitals supported by a telestroke network had higher rates of stroke care according to OPS (51.5% vs. 28.7%; *p* < 0.001), while rates of transfer to another hospital (7,8% vs. 22.7%; *p* < 0.001) and discharge to a nursing facility (2,6% vs. 8,0%; *p* < 0.007) were lower.

Costs per patient with related transfers were also calculated for the second comparison of model 1. The Mann–Whitney-U test showed significantly lower median costs for patients from hospitals with support by a telestroke network (model 1: 4,452€ vs. 5,924€; *p* = 0.002).

## Discussion

In our analysis, we showed that hospitals supported by a telestroke network were able to provide qualified stroke care in a significant number of cases between 2018 and 2021, while rates of stroke care according to OPS and rates of IVT improved over years. Significant differences were seen in both comparative approaches: Patients from hospitals with support by a telestroke network had a higher rate of stroke care according to OPS 8-98b, a higher IVT rate and a lower rate of secondary transfers to another hospital. Of note, length of stay and transfer to another hospital were independently associated with higher costs, while teleneurology despite the higher quality in stroke treatment was not.

Krogias et al. (2020) extracted the proportions of ischemic strokes in Germany treated with stroke care according to OPS 8-981 or 8-98b for the years 2012 to 2018 [14]. They showed that in 2018, 73.7% of all cases in Germany received stroke care and in Lower Saxony a proportion of 75% was achieved, which is higher than in our analysis in hospitals with support by telestroke networks. Since in hospitals supported by a telestroke network the level of stroke care according to OPS 8-98b is suggested to build up over time, the achieved rate is still considerable. Therefore, future steps consist of long-term support of hospitals supported by a telestroke network so that the criteria of stroke care according to 8-98b could be permanently fulfilled with potentially increasing rates.

In our study, the IVT rate in patients from hospitals supported by a telestroke network that was pictured over time increased steadily during the observation period. In model 1, IVT rates were higher in patients from hospitals supported by a telestroke network. The same applies for model 2, but here no significant difference was found, probably due to the small sample size. In both models, there was a clear difference between the groups, as no IVT was performed in the comparison groups of hospitals or districts without support by a telestroke network. Krogias et al. (2020) determined IVT rates for Germany in a nationwide approach in addition to the rate of stroke care according to OPS [14]. For 2018, the authors reported a nationwide IVT rate of 16.4% and a 15.8% rate for Lower Saxony. Barlinn et al. (2021) examined 17 teleneurology networks in Germany in 2018 and determined an IVT rate of 14.1% [13]. Müller-Barna et al. (2014) evaluated one of the first telestroke networks to be set upon the basis of routine data from a statutory health insurance scheme [20]. The study also documented an increasing IVT rate from 2.6% in 2003 to 15.5% in 2012. The authors argue that the higher IVT rate has led to better care as achieved in hospitals with support by a telestroke network. In our sample, the IVT rate rose steadily from 2018 to 2021 with a rate of 13.2% in 2021.

Another relevant result in both comparative approaches is that hospitals with support by a telestroke network had a significantly lower proportion of cases transferred to another hospital assuming a more qualified stroke care. Lyerly et al. (2022) analysed the impact of teleneurology network implementation on case referrals before and after implementation with an amount of 3,488 stroke encounters [21]. In their analysis the implementation of a telestroke network led to a reduction in transferred cases and hospitals with a lower annual number of strokes were more likely to transfer cases. Importantly, in our comparison lower numbers per hospital also occurred in those hospitals which were not supported by telestroke networks, and of these, a significant proportion of patients were transferred, presumably due to a lack of treatment options at the individual hospital. The necessity of transfers represents a risk both medically for the patient and economically for the health insurer. Providing care closer to home can close gaps in care and prevent unnecessary onward transfers. Similar results were found in the evaluation of a telestroke network in Catalonia [22]. López-Cancio et al. (2018) found that onward transfers were prevented after the implementation of a telestroke network through telestroke consultation in 46.8% of all ischemic strokes, 76.5% of all TIAs and 23.5% of all intracerebral hemorrhages. A hospital without appropriate stroke care has to decide in the case of a suspected acute stroke whether to treat the patient in the hospital or to transfer the patient to a specialized stroke center within a specific distance. This could result in duplicate cases that would possibly no longer occur in hospitals supported by telestroke networks and could also be cost-saving as our results for both models indicate.

In hospitals supported by a telestroke network, qualified stroke care is achieved with examinations and therapies, such as IVT leading to stroke care according to OPS. Lazarus et al. (2020) found that telestroke implementation especially in rural areas has led to better clinical outcomes compared to hospitals with no stroke-specific care [23]. However, the qualified stroke care also needs reimbursement accordingly. Importantly, despite all these measures, our findings in univariate analysis show that costs per patients’ case are lower in hospitals supported by a telestroke network. To verify whether the costs are indeed lower, we conducted a multiple regression analysis, revealing that length of stay and transfer to another hospital are independently associated with higher costs per patients, while teleneurological treatment is not. Consequently, teleneurological treatment does not lead to higher or lower costs. Instead, it appears that transfers to another hospital result in an average cost increase in the multiple four-digit euro range. Furthermore, we have already shown that the rate of transfers is increased in hospitals without support by a telestroke network. The analysis considered only inpatient care costs so that total costs are probably underestimated, since follow-up treatments and corresponding future costs are also reduced by improved patient care. It may be assumed, that better outcomes in high-quality treatment have also an effect on overall costs.

## Limitations

We included insurance data from the Lower Saxony TK resulting in a large number of cases available. However, we used routine data which are collected solely for billing purposes and not for scientific analysis. Accordingly, the data do not include many clinical values (e.g., laboratory or imaging results), in-hospital medication or non-billable services. Furthermore, we cannot exclude the possibility of a selection bias because we only included patients insured by the TK and diagnosed with a stroke or TIA. However, persons insured with TK are representative with respect to the German population. In addition, there is no information about patients changing their health insurance providers. Nevertheless, the number of people insured by TK has hardly changed in the analysed years. Furthermore, mainly older persons, especially after suffering from a stroke, are not expected to change their providers quite frequently. Finally, the validity of the routine data depends on the documentation process itself resulting in possible information bias.

A further limitation is that the control hospitals show significantly lower numbers of stroke cases than the hospitals supported by a telestroke network and we are therefore unable to establish an optimized group balance and perform a matching approach in the models. This is certainly due to the fact that the regional allocation concepts of the emergency services already take into account the inadequate stroke care in the respective hospitals. Hospitals without a care concept for acute stroke patients are less likely to be referred to directly using EMS bypass systems e.g. for suspected LVO. Model 2 attempts to reflect the highest possible rate of direct referrals, as there are no neurological stroke units in the districts under consideration. At this point, it is important to point out that the longer transportation times taken by the emergency services as a result have a negative impact on patient outcome according to the time is brain principle. Despite the aforementioned weakness in our models, an IVT rate of 0% and a transfer rate of almost 30% in the control hospitals represent clear results and strongly indicate care deficits that could be covered by support from telestroke networks. However, low patient numbers for this subgroup prohibited a multivariate analysis to test an independent association between IVT and teleneurological treatment.

During our analysis, the Covid-19 pandemic took place and could have had an impact on our results. Several studies have shown that the lockdowns in the Covid-19 pandemic led to a lower number of admitted stroke patients in the hospitals and lower rates of IVT treatment at least in the first months of the pandemic [24–26]. But, these were short-term trends, which should play a minor role in the multi-year period under observation.

## Conclusion

Teleneurology can improve stroke care. This paper suggests that hospitals with support by a telestroke network can provide qualified stroke care resulting in higher IVT rates, higher rates of qualified stroke care according to OPS and lower rates of onward transfers, which are independently associated with higher costs. Demographic change and ongoing urbanisation will pose challenges in how to provide high quality stroke care, especially in rural areas. Telestroke networks can be an option to meet these challenges.

## Supplementary Information

Supplementary Material 1: Appendix. Table A1. Indicators for hospital selection. Table A2. OPS codes. Table A3. Comparison of stroke cases in hospitals with or without support by a telestroke network between 2018 and 2021. Table A4. Comparison of costs of stroke cases in hospitals with or without support by a telestroke network between 2018 and 2021.

## Data Availability

The datasets used and analysed in the study are not publicly available but may be available from the techniker krankenkasse on reasonable request. Access to the data was obtained from the techniker krankenkasse under data disclosure agreements.

## Abbreviations

AIS: Acute ischemic stroke
CSC: Comprehensive Stroke Center
DRG: Diagnosis-Related Group
EVT: Endovascular treatment
GDP: Gross domestic product
ICD: International Classification Diseases
ICH: Intracranial hemorrhage
IVT: Intravenous thrombolysis
MHH: Hannover Medical School
MHH TNN: Hannover Medical School Teleneurology Network
OPS: Operation and Procedure Code
SU: Stroke Unit
TIA: Transient ischemic attack
TK: Techniker Krankenkasse
