# Supplementary material for: Support by telestroke networks is associated with increased intravenous thrombolysis and reduced hospital transfers: A german claims data analysis
Source: Health Econ Rev. 2024 Nov 28;14:100. doi: 10.1186/s13561-024-00577-5 (PMC11603936; doi:10.1186/s13561-024-00577-5)
Supplement: Supplementary file 1 — Supplementary Material 1: Appendix. Table A1. Indicators for hospital selection. Table A2. OPS codes. Table A3. Comparison of stroke cases in hospitals with or without support by a telestroke network between 2018 and 2021. Table A4. Comparison of costs of stroke cases in hospitals with or without support by a telestroke network between 2018 and 2021. [file 13561_2024_577_MOESM1_ESM.docx]

**Supplementary Material**

**Appendix**

**Table A1. Indicators for hospital selection**

| **Indicators** |
| --- |
| Location [18] |
| Number of internal medicine beds[18] |
| Number of neurological beds[18] |
| Number of total beds [18] |
| Support by a telestroke network |
| Neurological department [18] |
| Distance to next certified Stroke Unit |
| Distance to next comprehensive stroke center |
| Case numbers with stroke diagnoses according to quality report in 2016 – 2020 [19] |

**Table A2. OPS codes**

| **OPS Codes** | **Description** |
| --- | --- |
| 8-980 | Intensive care complex treatment (basic procedure) |
| 8-98f | Complex intensive care treatment (basic procedure) |
| 8-981 | Neurological complex treatment of acute stroke |
| 8-98b | Other neurological complex treatment of acute stroke |
| 8-020.8 | Systemic thrombolysis |

**Sensitivity Analysis: Comparison of stroke cases in hospitals with or without support by a telestroke network between 2018 and 2021**

**Table A3. Comparison of stroke cases in hospitals with or without support by a telestroke network between 2018 and 2021**

|  | **Model 1** | | |
| --- | --- | --- | --- |
|  | **(1)**  **(n=346)** | **(2)**  **(n=136)** | ***p-value*** |
| Age [in years], median (IQR) | 75 (62-82) | 77 (68-82) | 0.130 |
| Length of stay [in days], median (IQR) | 5 (3-7) | 5 (2-14) | 0.367 |
| DRG Weight, median (IQR) | 0.94 (0.74-1.19) | 0.94 (0.59-1.31) | 0.857 |
| Ventilation hours [in hours], median (IQR) | 0 | 0 | 0.240 |
| PCCL, median (IQR) | 0 (0-1) | 0 (0-2) | **0.008** |
| OPS 8-98b^a^ (%) | 51.5 | 28.7 | **<0.00**1 |
| OPS in total^a^ (%) | 72.0 | 31.6 | **<0.001** |
| Death^a^ (%) | 3.1 | 3.6 | 0.784 |
| Transfer to another hospital^a^ (%) | 7.8 | 22.7 | **<0.001** |
| Discharge to a rehabilitation facility^a^ (%) | 4.3 | 5.8 | 0.474 |
| Discharge to a nursing facility^a^ (%) | 2.6 | 8.0 | **0.007** |

Continuous data are presented as median (interquartile range) and discrete data as proportions. Statistically significant results are shown in bold, p<0.05 was considered significant. OPS 8-98b includes all cases with documented OPS code 8-98b, OPS in total includes all cases with documented OPS codes 8-980, 8-98f, 8-981, 8-98b. Death, transfer to another hospital, discharge to a rehabilitation facility and discharge to a nursing facility describe reasons for discharge that have been documented for each case.

*Abbreviation*: IQR, Interquartile range. OPS, Operation and procedure code, PCCL, Patient clinical complexity level. Group description: (1)=Hospitals supported by a telestroke network (2)=Hospitals not supported by a telestroke network.

**Table A4. Comparison of costs of stroke cases in hospitals with or without support by a telestroke network between 2018 and 2021**

|  | **Model 1** | | |
| --- | --- | --- | --- |
|  | **(1)**  **(n=346)** | **(2)**  **(n=136)** | ***p-value*** |
| Costs [in Euro], median (IQR)  mean (SD) | 4,452 (3,524-6,338)  6,027 (6,023) | 5,924 (3,616-9,785)  8,923 (11,475) | **0.002** |

Data are presented as median (interquartile range) and mean (standard deviation). Statistically significant results are shown in bold, p<0.05 was considered significant.

*Abbreviation*: IQR. Interquartile range, Group description: (1)=Hospitals supported by a telestroke network in districts without certified SU, (2)=Hospitals without support by a telestroke network in districts without SU.
